# Molecular Investigation of Recurrent *Streptococcus iniae* Epizootics Affecting Coral Reef Fish on an Oceanic Island Suggests at Least Two Distinct Emergence Events

**DOI:** 10.3389/fmicb.2021.749734

**Published:** 2021-11-04

**Authors:** Solène Irion, Oleksandra Rudenko, Michael Sweet, Pascale Chabanet, Andrew C. Barnes, Pablo Tortosa, Mathieu G. Séré

**Affiliations:** ^1^Université de La Réunion, Unité Mixte de Recherche, Processus Infectieux en Milieu Insulaire Tropical (UMR PIMIT), Inserm1187, CNRS9192, IRD249, Plateforme de Recherche CYROI, Saint Denis, France; ^2^Université de La Réunion, Unité Mixte de Recherche, Ecologie marine tropicale des océans Pacifique et Indien (UMR ENTROPIE), CNRS, IRD, Saint Denis, France; ^3^School of Biological Sciences, Centre for Marine Science, The University of Queensland, Brisbane, QLD, Australia; ^4^Aquatic Research Facility, Environmental Sustainability Research Centre, University of Derby, Derby, United Kingdom

**Keywords:** *Streptococcus iniae*, MLST, epizootics, reef fish, aquaculture

## Abstract

*Streptococcus iniae* is an emerging zoonotic pathogen of increasing concern for aquaculture and has caused several epizootics in reef fishes from the Caribbean, the Red Sea and the Indian Ocean. To study the population structure, introduction pathways and evolution of *S. iniae* over recurring epizootics on Reunion Island, we developed and validated a Multi Locus Sequence Typing (MLST) panel using genomic data obtained from 89 isolates sampled during epizootics occurring over the past 40years in Australia, Asia, the United States, Israel and Reunion Island. We selected eight housekeeping loci, which resulted in the greatest variation across the main *S. iniae* phylogenetic clades highlighted by the whole genomic dataset. We then applied the developed MLST to investigate the origin of *S*. *iniae* responsible for four epizootics on Reunion Island, first in inland aquaculture and then on the reefs from 1996 to 2014. Results suggest at least two independent *S*. *iniae* emergence events occurred on the island. Molecular data support that the first epizootic resulted from an introduction, with inland freshwater aquaculture facilities acting as a stepping-stone. Such an event may have been facilitated by the ecological flexibility of *S. iniae*, able to survive in both fresh and marine waters and the ability of the pathogen to infect multiple host species. By contrast, the second epizootic was associated with a distinct ST of cosmopolitan distribution that may have emerged as a result of environment disturbance. This novel tool will be effective at investigating recurrent epizootics occurring within a given environment or country that is despite the fact that *S. iniae* appears to have low genetic diversity within its lineage.

## Introduction

Streptococcal infections underlie disease outbreaks in numerous farmed and wild fish species, causing septicemia, central nervous system damage and meningoencephalitis (Eldar et al., 1994; Toranzo et al., 2005). The pathologic agent, *Streptococcus iniae* has been directly linked to massive economic losses in both marine and freshwater aquaculture environments, with mortality rates reaching 75% in tilapia farms for example (Perera et al., 1994; Eldar et al., 1997a; Francis et al., 2014). In addition, *S. iniae* has been known to infect mammals as well, such as dolphins (Pier and Madin, 1976; Agnew and Barnes, 2007; Song et al., 2017) and is occasionally zoonotic, generating soft tissue infections and sepsis in humans (Weinstein et al., 1997; Koh et al., 2004; Lau et al., 2006).

Although first isolated in the 1970s (from abscesses in captive freshwater dolphins), the first recorded *S. iniae* outbreaks in farmed fish were documented in the 1980s throughout Japan, the United States, Israel, Australia and Asia (Eldar et al., 1994; Perera et al., 1994; Stoffregen et al., 1996; Bromage et al., 1999; Nguyen et al., 2002). Since then, the geographical range of these epidemics has expanded with cases being reported throughout Europe, South America, the Middle East and Africa (El Aamri et al., 2010; Fadaeifard et al., 2011; Figueiredo et al., 2012; Türe and Alp, 2016). Inactivated (or killed) vaccines were developed in 1995 in order to mitigate the economic consequences of these outbreaks (Eldar et al., 1997a; Klesius et al., 2000; Shoemaker et al., 2010). However, the efficacy of vaccination has been challenged as reinfection of vaccinated stock are known to occur, most notably following the emergence of new serotypes bypassing vaccine protection through spontaneous point mutations in genes involved in capsule biosynthesis (Bachrach et al., 2001; Eyngor et al., 2008; Millard et al., 2012; Barnes and Silayeva, 2016).

Although epidemics are usually associated with farmed fish, several outbreaks have been reported in wild populations. Whilst the majority of these were in the vicinity of aquaculture facilities (Zlotkin et al., 1998; Colorni et al., 2002), a result suggesting possible transmission between cultured and wild fish, there are a number of instances where recurrent mass mortalities of reef fish have occurred in the absence of such connection (Ferguson et al., 2000; Keirstead et al., 2014). For example, mass mortalities of reef fish have occurred in 2002 and 2014 on Reunion Island (an overseas department of France and a geographically isolated oceanic island located in the western Indian Ocean; Turquet et al., 2002; Quod et al., 2014). Several fish kill phenomena have been reported during the austral summer from 2001 to 2003 (Pothin et al., 2001; Turquet et al., 2002) and more recently between January and May of 2014 (Quod et al., 2014). The last recorded episode (2014) was the most virulent with thousands of fish killed, including 34 families such as Surgeon fish (Acanthuridae, 12 sp.), Triggerfish (Balistidae, 8 sp.) and Groupers (Serranidae, 6 sp.).

Although the fish kills were linked to *S. iniae* infection, transmission of the pathogen was unknown, specifically regarding the origin of the causal agent. Molecular epidemiology is often employed to identify the transmission chains of specific pathogens within a given environment. For *S. iniae*, the diversity and evolutionary history of specific isolates has been assessed using molecular fingerprinting techniques. For example, restriction fragment length polymorphism was used to distinguish isolates responsible for outbreaks in American and Israeli fish farms (Eldar et al., 1997b, 1999). Whilst, other studies focused on using random amplified polymorphic DNA (Dodson et al., 1999; Kvitt and Colorni, 2004; Erfanmanesh et al., 2012), however both methods showed marginal discriminatory power. Pulsed-field gel electrophoresis (PFGE) has also been employed to evaluate strain diversity in *S. iniae* and shows the greatest discriminative power being able to differentiate between pathogenic and commensal strains (Fuller et al., 2001). PFGE also allows for discrimination between isolates collected within a specific region over a short period of time (Nawawi et al., 2008; Zhou et al., 2008), and strains collected during different epizootics in several host species (Facklam et al., 2005; Nawawi et al., 2008). However, PFGE does not necessarily address the phylogenetic history of the bacteria, as it targets a highly variable region within the microbial genome (Achtman, 2008). Further, PFGE appears to be poorly reproducible from one laboratory to the other and this lack of data transferability impedes analyses at a global scale. Therefore, we sought to explore other options to allow us to understand the potential point of origin of the *S. iniae* involved in the fish die offs in Reunion.

Multi Locus Sequence Typing (MLST) is a robust and reproducible method that has been widely used to characterize bacterial isolates since its introduction in 1998 (Maiden et al., 1998). It is based on the sequencing of several housekeeping genes in order to characterize a strain through a unique combination of alleles corresponding to an arbitrary sequence type (ST) number (Maiden, 2006). In the era of genomics, MLST is still considered relevant since it provides the overall clonal frame, population structure and diversity of a bacterial taxa (Pérez-Losada et al., 2013). Here, we therefore aimed to develop the first MLST scheme for *S. iniae* using eight selected housekeeping genes to identify possible origins of four wild and farmed fish epizootics, which have occurred in Reunion from 1996 onwards.

## Materials and Methods

### Bacterial Strains

**Figure 1:**
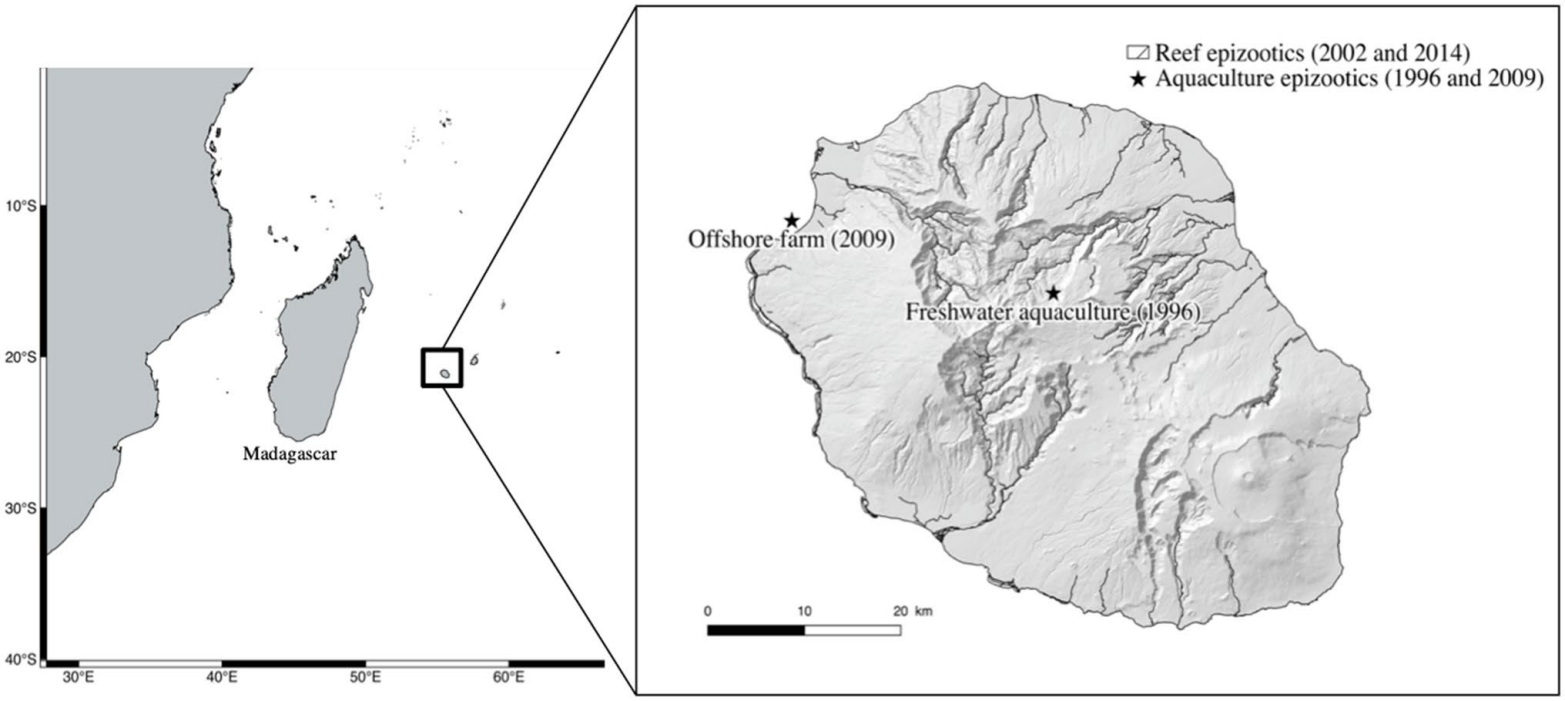
Map of the epizootics investigated on Reunion Island since 1996. Reef epizootics in 2002 and 2014 were spread along the West coast of the island on different coral reefs.

Three Reunionese strains of *S. iniae* were utilized in this study. The first was isolated in 2002 from a grouper (*Variola louti*). The second was isolated in 2014 from a striped large-eye bream (*Gnathodentex aurolineatus*) and the third, isolated in 2009 from diseased red drum (*Sciaenops ocellatus*). This last isolate was attributed as the cause of a fish kill in an offshore marine fish farm (Table 1). In addition, we utilized the type strain CIP 103769^T^. Figure 1 pinpoints the location of the epizootics investigated on Reunion Island.

**Table 1:** Origin and typing of *S. iniae* strains. For Australian strains, region of isolation is indicated.

Accession nb. / Strain	Host species	Environment	Year of first isolation	Region	ST^a^	CC^b^	Allelic profile^c^
CP005941.1	*Paralichthys olivaceus* (flounder)	SW, farm	2006	China	4	1	1,1,1,1,1,1,1,1
CP007586.1	*Oreochromis sp*. (tilapia)	FW, farm	2005	Israel	13	1	1,1,3,1,1,5,1,1
CP007587.1	*Oreochromis sp*. (tilapia)	FW, farm	2005	Israel	13	1	1,1,3,1,1,5,1,1
CP010783.1	*Paralichthys olivaceus* (flounder)	SW, farm	2012	South Korea	3	1	1,1,1,2,1,1,1,1
CP017952.1	*Oreochromis sp*. (tilapia)	FW, farm	2000	Taiwan	4	1	1,1,1,1,1,1,1,1
QMA0071	*Lates calcarifer* (barramundi)	FW, farm	2000	QLD	4	1	1,1,1,1,1,1,1,1
QMA0074	*Lates calcarifer* (barramundi)	FW, farm	1998	QLD	7	NA	2,1,1,1,1,2,1,1
QMA0077	*Lates calcarifer* (barramundi)	FW, farm	1995	QLD	7	NA	2,1,1,1,1,2,1,1
QMA0078	*Lates calcarifer* (barramundi)	FW, farm	2001	QLD	4	1	1,1,1,1,1,1,1,1
QMA0080	*Lates calcarifer* (barramundi)	FW, farm	2004	WA	8	1	3,1,1,1,1,1,1,1
QMA0082	*Lates calcarifer* (barramundi)	FW, farm	2004	WA	8	1	3,1,1,1,1,1,1,1
QMA0083	*Lates calcarifer* (barramundi)	FW, farm	2004	WA	4	1	1,1,1,1,1,1,1,1
QMA0084	*Epalzeorhynchos kalopterus* (flying fox fish)	aquarium	2001	WA	6	NA	1,2,1,1,1,3,1,1
QMA0087	*Lates calcarifer* (barramundi)	FW, farm	2004	WA	4	1	1,1,1,1,1,1,1,1
QMA0130	*Homo sapiens*		1995	Canada	2	1	1,1,1,2,2,1,1,1
QMA0131	*Homo sapiens*		1995	Canada	2	1	1,1,1,2,2,1,1,1
QMA0133	*Homo sapiens*		2001	USA	3	1	1,1,1,2,1,1,1,1
QMA0134	*Homo sapiens*		2001	USA	3	1	1,1,1,2,1,1,1,1
QMA0135	*Homo sapiens*		2002	USA	3	1	1,1,1,2,1,1,1,1
QMA0137	*Homo sapiens*		2004	USA	3	1	1,1,1,2,1,1,1,1
QMA0138	*Homo sapiens*		2004	USA	3	1	1,1,1,2,1,1,1,1
QMA0139	fish (unknown sp.)	NA	1996	Canada	9	2	1,3,1,1,1,1,1,2
QMA0140	*Inia geoffrensis* (Amazon freshwater dolphin)	aquarium	1976	USA	4	1	1,1,1,1,1,1,1,1
QMA0141	*Inia geoffrensis* (dolphin)	aquarium	1978	USA	1	NA	4,4,2,3,3,4,2,3
QMA0142	*Lates calcarifer* (barramundi)	SW, farm	2005	NT	8	1	3,1,1,1,1,1,1,1
QMA0150	*Lates calcarifer* (barramundi)	SW, farm	2005	NT	8	1	3,1,1,1,1,1,1,1
QMA0155	*Lates calcarifer* (barramundi)	FW, farm	2005	NSW	4	1	1,1,1,1,1,1,1,1
QMA0156	*Lates calcarifer* (barramundi)	FW, farm	2005	NSW	4	1	1,1,1,1,1,1,1,1
QMA0157	*Lates calcarifer* (barramundi)	FW, farm	2005	NSW	4	1	1,1,1,1,1,1,1,1
QMA0158	*Lates calcarifer* (barramundi)	FW, farm	2006	SA	4	1	1,1,1,1,1,1,1,1
QMA0159	*Lates calcarifer* (barramundi)	FW, farm	2006	SA	4	1	1,1,1,1,1,1,1,1
QMA0160	*Lates calcarifer* (barramundi)	FW, farm	1999	SA	4	1	1,1,1,1,1,1,1,1
QMA0161	*Lates calcarifer* (barramundi)	FW, farm	2000	SA	4	1	1,1,1,1,1,1,1,1
QMA0162	*Lates calcarifer* (barramundi)	FW, farm	2000	SA	4	1	1,1,1,1,1,1,1,1
QMA0163	*Lates calcarifer* (barramundi)	FW, farm	2000	SA	4	1	1,1,1,1,1,1,1,1
QMA0164	*Lates calcarifer* (barramundi)	FW, farm	2006	QLD	7	NA	2,1,1,1,1,2,1,1
QMA0165	*Lates calcarifer* (barramundi)	FW, farm	2006	QLD	7	NA	2,1,1,1,1,2,1,1
QMA0177	*Lates calcarifer* (barramundi)	SW, farm	2006	NT	8	1	3,1,1,1,1,1,1,1
QMA0180	*Lates calcarifer* (barramundi)	SW, farm	2006	NT	8	1	3,1,1,1,1,1,1,1
QMA0186	*Oncorhynchus mykiss* (rainbow trout)	FW, farm	2000	Israel	11	1	1,1,3,1,1,1,1,1
QMA0187	*Channa striata* (snakehead fish)	NA	1983	Thailand	5	NA	1,1,4,1,1,1,3,1
QMA0188	*Oncorhynchus mykiss* (rainbow trout)	FW, farm	1998	Israel	11	1	1,1,3,1,1,1,1,1
QMA0189	*Oncorhynchus mykiss* (rainbow trout)	FW, farm	1996	Reunion	11	1	1,1,3,1,1,1,1,1
QMA0190	*Channa striata* (snakehead fish)	NA	1988	Thailand	9	2	1,3,1,1,1,1,1,2
QMA0191	*Lates calcarifer* (barramundi)	SW, farm	2005	NT	8	1	3,1,1,1,1,1,1,1
QMA0207	*Lates calcarifer* (barramundi)	SW, farm	2006	NT	8	1	3,1,1,1,1,1,1,1
QMA0216	*Lates calcarifer* (barramundi)	FW, farm	2007	QLD	4	1	1,1,1,1,1,1,1,1
QMA0218	*Lates calcarifer* (barramundi)	FW, farm	2007	QLD	7	NA	2,1,1,1,1,2,1,1
QMA0220	*Lates calcarifer* (barramundi)	FW, farm	2006	NSW	4	1	1,1,1,1,1,1,1,1
QMA0221	*Lates calcarifer* (barramundi)	FW, farm	2007	NSW	4	1	1,1,1,1,1,1,1,1
QMA0222	*Lates calcarifer* (barramundi)	FW, farm	2006	NSW	4	1	1,1,1,1,1,1,1,1
QMA0233	*Lates calcarifer* (barramundi)	FW, farm	2009	NSW	10	2	1,3,1,4,1,1,1,2
QMA0234	*Lates calcarifer* (barramundi)	FW, farm	2009	NSW	10	2	1,3,1,4,1,1,1,2
QMA0235	*Lates calcarifer* (barramundi)	FW, farm	2009	NSW	10	2	1,3,1,4,1,1,1,2
QMA0236	*Lates calcarifer* (barramundi)	FW, farm	2009	NSW	10	2	1,3,1,4,1,1,1,2
QMA0244	*Lates calcarifer* (barramundi)	FW, farm	2008	SA	4	1	1,1,1,1,1,1,1,1
QMA0245	*Lates calcarifer* (barramundi)	FW, farm	2008	SA	4	1	1,1,1,1,1,1,1,1
QMA0246	*Lates calcarifer* (barramundi)	FW, farm	2009	SA	4	1	1,1,1,1,1,1,1,1
QMA0247	*Lates calcarifer* (barramundi)	FW, farm	2009	SA	4	1	1,1,1,1,1,1,1,1
QMA0248	*Lates calcarifer* (barramundi)	FW, farm	2009	SA	4	1	1,1,1,1,1,1,1,1
QMA0249	*Lates calcarifer* (barramundi)	FW, farm	2009	SA	10	2	1,3,1,4,1,1,1,2
QMA0250	*Lates calcarifer* (barramundi)	FW, farm	2007	NSW	4	1	1,1,1,1,1,1,1,1
QMA0251	*Lates calcarifer* (barramundi)	FW, farm	2008	NSW	4	1	1,1,1,1,1,1,1,1
QMA0252	*Lates calcarifer* (barramundi)	FW, farm	2008	NSW	4	1	1,1,1,1,1,1,1,1
QMA0253	*Lates calcarifer* (barramundi)	FW, farm	2009	NSW	10	2	1,3,1,4,1,1,1,2
QMA0254	*Lates calcarifer* (barramundi)	FW, farm	2009	NSW	10	2	1,3,1,4,1,1,1,2
QMA0258	*Lates calcarifer* (barramundi)	FW, farm	2008	QLD	4	1	1,1,1,1,1,1,1,1
QMA0371	*Scortum barcoo* (jade perch)	FW, farm	2011	NSW	4	1	1,1,1,1,1,1,1,1
QMA0373	*Lates calcarifer* (barramundi)	FW, farm	2012	QLD	7	NA	2,1,1,1,1,2,1,1
QMA0374	*Lates calcarifer* (barramundi)	FW, farm	2012	QLD	7	NA	2,1,1,1,1,2,1,1
QMA0445	*Oreochromis sp*. (tilapia)	FW, farm	1998	USA	4	1	1,1,1,1,1,1,1,1
QMA0446	*Oreochromis sp*. (tilapia)	FW, farm	1998	USA	3	1	1,1,1,2,1,1,1,1
QMA0447	*Morone chrysops × Morone saxatilis* (hybrid striped bass)	FW, farm	1996	USA	3	1	1,1,1,2,1,1,1,1
QMA0448	*Morone chrysops × Morone saxatilis* (hybrid striped bass)	FW, farm	1998	USA	4	1	1,1,1,1,1,1,1,1
QMA0457	*Oreochromis sp*. (tilapia)	FW, farm		USA	4	1	1,1,1,1,1,1,1,1
QMA0458	*Epalzeorhynchos bicolor* (Redtail sharkminnow)	Ornamental aquaria	2004	USA	4	1	1,1,1,1,1,1,1,1
QMA0462	*Chromobotia macracanthus* (botia)	Ornamental aquaria	2005	USA	6	NA	1,2,1,1,1,3,1,1
QMA0463	*Chromobotia macracanthus* (botia)	Ornamental aquaria	2005	USA	6	NA	1,2,1,1,1,3,1,1
QMA0466	*Oreochromis sp*. (tilapia)	FW, farm	2005	USA	2	1	1,1,1,2,2,1,1,1
QMA0467	*Epalzeorhynchos frenatum*	Ornamental aquaria	2004	USA	4	1	1,1,1,1,1,1,1,1
QMA0468	*Oreochromis sp*. (tilapia)	FW, farm	2005	USA	4	1	1,1,1,1,1,1,1,1
QMA0490	*Oreochromis sp*. (tilapia)	FW, farm	2015	Honduras	4	1	1,1,1,1,1,1,1,1
QMA0491	*Oreochromis sp*. (tilapia)	FW, farm	2015	Honduras	4	1	1,1,1,1,1,1,1,1
QMA0492	*Oreochromis sp*. (tilapia)	FW, farm	2015	Honduras	4	1	1,1,1,1,1,1,1,1
QMA0493	*Oreochromis sp*. (tilapia)	FW, farm	2016	Honduras	4	1	1,1,1,1,1,1,1,1
CIP-103769	*Oreochromis sp*. (tilapia)	FW, farm	1989	Israel	11	1	1,1,3,1,1,1,1,1
RUN_2002	*Variola louti* (grouper)	Reef	2002	Reunion	11	1	1,1,3,1,1,1,1,1
RUN_2009	*Sciaenops ocellatus* (red drum)	SW, cages	2009	Reunion	12	1	1,5,3,1,1,1,1,1
RUN_2014	*Gnathodentex aurolineatus* (Striped large-eye bream)	Reef	2014	Reunion	4	1	1,1,1,1,1,1,1,1

*QLD: Queensland; NT: Northern Territory; NSW: New South Wales; SA: South Australia; WA: Western Australia; SW, seawater; FW, Freshwater*.

a *ST=Sequence Type, corresponding to an unique combination of alleles*.

b *CC=clonal complex, defined as a group of ST linked as single-locus variants to at least another ST within the CC*.

c *genes order=dnaN, mutL, mutM, mutS, mutX, recD2, rnhC, yfhQ*.

A further 85*S. iniae* genomes, (76 obtained from fish, two from dolphins, and seven from humans), representing all sequencing data available to date, were also used to assess the performance of the MLST for identifying origins of the three Reunionese strains. The sequences were available on Genbank (genome accession numbers in Table 1) or provided by co-authors (AB and OS). These isolates were temporally (isolated between 1976 and 2016) and geographically diverse, obtained from different countries including North and Central America, Australia, Asia and Israel (Table 1). The collection of isolates has been previously confirmed as belonging to *S. iniae* using the specific PCR protocol described by (Mata et al., 2004). Details regarding the origin of each isolate are provided in Table 1.

### Multi Locus Sequence Typing Development

#### Loci Selection

To design the Multi Locus Sequence Typing panel, 80 draft genomes assembled by co-authors (OS and AB) as part of a separate study (Silayeva et al., 2020) along with five published assembled genomes (available on Genbank) were aligned. Eight *loci* or housekeeping genes, including *dnaN* (encoding DNA polymerase III), *rnhC* (encoding Ribonuclease HIII), *yfhQ* (encoding an A/G-specific adenine glycosylase), *recD2* (encoding DNA helicase), *mutM* (encoding the formamidopyrimidine-DNA glycosylase), *mutX* (encoding the 8-oxo-dGTP diphosphatase), *mutL* and *mutS* (both encoding DNA mismatch repair proteins) were selected for MLST analysis. They were chosen because mutations in DNA repair *loci* are tightly linked to evolutionary history of organisms and, as such, they constitute promising genes for typing bacterial strains (Stich et al., 2010). For *S. iniae*, a previous study based on draft genomes indicated that these eight *loci* were correlated with phylogenetic diversification but highly conserved within phylogenetic clades, making them ideal candidates for the development of a MLST (Silayeva et al., 2020). Primer 3 (Untergasser et al., 2012) was then used to design primer pairs for each locus (Table 2).

**Table 2:** Primer sequences and genetic characteristics of the loci used for MLST.

Locus	Forward primer	Reverse primer	Amplified fragment (pb)	Coding region	Number of alleles	Number of polymorphic sites	H (*n*=89)^a^	dN-dS^b^
dnaN	GCACATGTTAATTCGCCAGAGG	CAGCACCAACTCTGATAATTTTCCA	404	[1–237][299–404]	4	4	0,3,164	−0,547
mutL	CCAACCAAGCAGGAAGTTCG	CGTTCTTGAGCTGCGTGTTG	545	[1–503]	5	9	0,2,814	−0,77
mutM	CAGAGTAGATGGTTTGACCC	TGCCCTGTATGATGCCTATC	410	[1–157]	4	5	0,2061	−0,358
mutS	TTTAACTGGCGCATCCCCAT	TGGATCTTGCAACAGGTGAGT	448	[1–448]	4	15	0,3,639	0,995
mutX	TGGCCATTGGTTTCATCAAGG	CGTAATCCCCTTCCCACGTT	547	[172–547]	3	4	0,0876	−1,553
recD2	AGGGCTTCCTAGTGCTACCA	ACTCGCTTTGCCCATCAAGA	563	[1–563]	5	6	0,2,658	−1,594
rnhC	GGAATCGCTGTTGTT GCAAGT	TTGAGTGTTTGCGAAGTGGC	582	[1–582]	3	4	0,0447	1,768
yfhQ	AGGCCAGGTGATTTCAACCA	CAGGAGAAACCCAGGCCATT	511	[1–511]	3	6	0,2040	−1,089

a *genetic diversity expressed as the probability that, at a single locus, any two alleles, chosen at random from the population, are different to each other*.

b *non-synonymous (d_N_)–synonymous substitutions (d_S_)*.

#### pcr

PCR conditions were tested on four strains for which no sequencing data existed, including the type strain CIP 103769^T^ and three Reunionese strains (collected between 2002 and 2014). The four strains used to test the PCR conditions were grown in Brain Heart Infusion Agar. Single colonies were placed in 100μl of 5mm Tris/HCL at 100°C for 5min. PCR were then run for each of the *loci* identified above. PCR mixture comprised 1μl of bacterial DNA, 12.5μl of MasterMix (Applied Biosystems, Foster City, United States) and 1μl of each primer (10μm) (Table 2 for the forward and reverse primers for each *loci*) in a total volume of 25μl. PCR conditions included an initial step of denaturation/lysis at 95°C for 5min, 30cycles of denaturation at 94°C for 30s, annealing at 56°C for 1min, and extension at 72°C for 2min, and a final step of extension at 72°C for 7min. Sanger sequencing (paired end reads) was performed by Genoscreen (Lille, France). Sequences were manually analyzed and trimmed to remove the primers using Geneious 10.1.3 (Kearse et al., 2012).

#### MLST Typing

The new sequences obtained for each MLST locus for these four strains were then compared with the sequences available from the five assembled genomes (available on Genbank) and 80 available partial genomes (Silayeva et al., 2020). Numbers were assigned arbitrarily to identify each distinct allele type (AT) at each locus. Each unique combination of AT defined a corresponding sequence type (ST), i.e., a group that shares the same combination of alleles at all eight *loci*. A clonal complex (CC) was defined as a group of ST linked as single-locus variants to at least another ST within the CC. Clonal complexes and evolutionary relationships between isolates were analyzed with a minimum spanning tree using the global optimal eBURST algorithm implemented in PHYLOViZ (Feil et al., 2004; Francisco et al., 2012).

### Data Analysis

The discriminatory power of the MLST scheme was measured using the Simpson’s index (Hunter and Gaston, 1988). This index measures the probability for two randomly sampled strains to present different STs. 95% confidence interval for this index was calculated using the Comparing Partitions website.^1^ The standardized index of association, I_A_^S^, was calculated both on the complete data set (89 strains) and on a subset representing each ST only once (13 STs) using LIAN 3.7 (Haubold and Hudson, 2000).^2^ I_A_^S^ is used to evaluate the role of recombination in population evolution. A value of zero corresponds to frequent recombination events while clonal populations are identified by an I_A_^s^ value significantly differing from zero (Smith et al., 1993). LIAN 3.7 was also used to calculate the genetic diversity (H) at each locus, analyzed as a measure of their expected genetic variability. This value ranges from 0 (no diversity) to 1. The number of variable nucleotide sites, non-synonymous (*d_N_*) and synonymous substitutions (*d_S_*) as well as Z test of selection on coding regions were calculated using Mega7 (Kumar et al., 2016). The *Z* test evaluates the null hypothesis of strict neutrality (*d_N_ =d_S_*) versus the alternative hypotheses of purifying (*d_N_ <d_S_*) or positive (*d_N_ >d_S_*) selection (Nei and Gojobori, 1986) and was performed on coding regions only, using 1,000 bootstrap replications and Jukes-Cantor adjustments.

### Accession Numbers of Sequences

DNA sequences of the alleles determined in this study for each locus were deposited on Genbank under the accession numbers MN327582 to MN327612. Accession numbers for each allele of the MLST are provided in Table 3. The scheme is curated and available to the public *via* pubMLST (Jolley et al., 2018) at: https://pubmlst.org/organisms/streptococcus-iniae/

**Table 3:** Accession number of the MLST scheme.

Locus	Allele number in the MLST	Accession number
dnaN	1	MN327582
	2	MN327583
	3	MN327584
	4	MN327585
MutL	1	MN327590
	2	MN327586
	3	MN327587
	4	MN327588
	5	MN327589
MutM	1	MN327591
	2	MN327592
	3	MN327593
	4	MN327594
MutS	1	MN327595
	2	MN327596
	3	MN327597
	4	MN327598
MutX	1	MN327599
	2	MN327600
	3	MN327601
recD2	1	MN327602
	2	MN327603
	3	MN327604
	4	MN327605
	5	MN327606
rnhC	1	MN327607
	2	MN327608
	3	MN327609
yfhQ	1	MN327610
	2	MN327611
	3	MN327612

## Results

### Locus Genetic Characteristics and Discriminatory Power of MLST

A total combined sequence length of 4,010 nucleotides was obtained when concatenating all eight housekeeping *loci*. Fragment lengths ranged from 404bp (*dna*N) to 582bp (*rnh*C). Considering all 89 strains, the number of alleles for a locus varied from three (*mut*X and *yfh*Q) to five (*rec*D2 and *mut*L) and the number of polymorphic sites from four (dnaN*, mut*X and *rnh*C) to 15 (*mut*S). The genetic diversity, estimated by the H index, reached a minimum of 0.0447 (*rnh*C) and a maximum of 0.3639 (*mut*S). The main characteristics of genetic diversity for each locus are summarized in Table 2.

For most genes, synonymous substitutions (*d_S_*) were more frequent than non-synonymous substitutions (*d_N_*). Only *mut*S and *rnh*C had more non-synonymous substitutions than synonymous substitutions but this result was not statistically significant (Table 2). The higher number of synonymous substitutions over non-synonymous substitutions could indicate that these loci are under neutral or purifying selection. However, the *Z* test for purifying selection was not significant for any loci. LIAN analysis of linkage disequilibrium based on the eight housekeeping genes suggested that the bacterial population analyzed in this study is in linkage disequilibrium when considering all 89 strains (I_A_^S^=0.1348; *p*<0.01), or a subset consisting of the 13 unique STs (I_A_^S^=0.1839; *p*<0.01). The discriminatory power of this MLST estimated by the Simpson’s index was 0.761 with a confidence interval of 95% between 0.677 and 0.845.

### Population Structure

**Figure 2:**
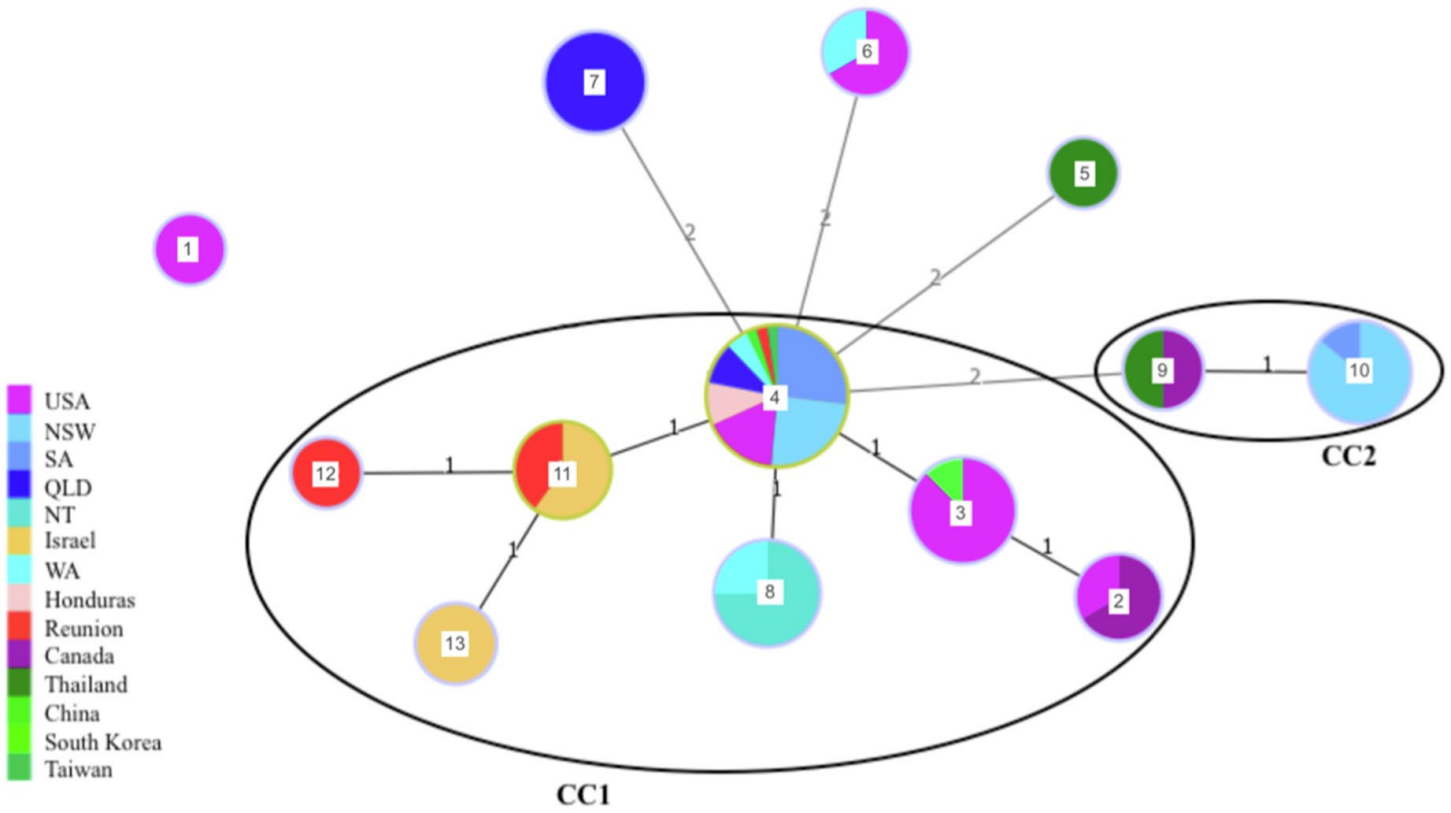
Minimum spanning tree of *S. iniae* collection illustrating the evolutionary relationships between isolates depending on their geographical origin. Each circle represents a ST and the size of the circle is proportional to the number of isolates within this ST (log scale). ST number is indicated at the center of each circle. Numbers over the connecting lines indicate the number of locus difference between the closest STs (1: SLV; 2: DLV). Clonal complexes are shown (7 of eight alleles shared).

The 89 strains analyzed in this study were grouped into 13 different sequence types (STs) and two clonal complexes (CCs; Figure 2). From the 13 STs, three (ST1, ST5 and ST12) were represented by a single isolate. ST1 corresponds to one of the non-fish hosts, an isolate obtained from a dolphin (*Inia geoffrensis*) in 1978. This ST was the most genetically distant and shared no alleles with any of the other 12 STs (Table 1 and Figure 2). ST5 was obtained from a giant snakehead (*Channa micropeltes*) isolated in Thailand in 1983 and ST12 from a red drum (*Sciaenops ocellatus*) in an offshore aquaculture facility in Reunion Island. The other STs comprised between two and 41 isolates each. CC1 was represented by sixty-eight isolates (76,40% of included isolates) and CC2 by nine isolates (23.60% of included isolates). Within CC1, 41 isolates (46.07% of the collection) belonged to ST4, which is considered by goeBURST analysis as the ancestral type or founder of CC1. As such, ST4 is the most common *S. iniae* ST in our collection and has been isolated from multiple geographical origins (United States, China, Taiwan, Australia, Reunion Island) over a large period of time (1976 to 2016). This ST has been found associated in our study to different fish species and a dolphin. CC2 comprised two distinct STs: ST10 (seven isolates) and ST9 (two isolates). ST10 is represented by vaccine escape strains sampled in Australian barramundi farms in 2009, considered as hypermutators (Barnes and Silayeva, 2016) whereas ST9 included the only Canadian isolate from fish and one of the two Thai isolates, again obtained from fish.

When the CC definition was relaxed (6/8 shared alleles), all STs except ST1 formed a single network (Figure 2) indicating close evolutionary relationships between the different STs. However, some STs seemed to be specific of a geographical area, such as ST7, which is found only in Queensland. Further, ST12 and ST13 have a limited geographical distribution (Reunion Island and Israel, respectively) and could be considered as descendants of ST11, which included earlier freshwater aquaculture isolates from these regions (trout and tilapia), as well as the first isolate involved in a reef epizootic in 2002 in Reunion Island. Altogether, despite a strong bias toward a few fish species, no clear host specificity could be detected, with isolates retrieved from mammals sharing STs isolated from fish as well (ST2, ST3, ST4, ST6). For instance, ST2 and ST3 were represented by fish and human isolates from North America, ST4 by fish and dolphin isolates, while ST6 included bat and ornamental fish isolates (Table 1).

## Discussion

### Discriminatory Power of the MLST Scheme

Considering the 89 strains tested within our study, the MLST scheme produced a Simpson’s Index of Diversity (SID) of 0.761, i.e., two strains randomly sampled in our collection have an average probability of 0.761 to display different STs. The genetic diversity estimated by the SID is well below 0.90, considered as the desirable threshold to interpret typing results with confidence (Hunter and Gaston, 1988). The observed lack of diversity in the *S. iniae* strains assed in this study is in keeping with other studies using MLST and indicates that it may be hard to find sufficient for highly-descriminatory MLST variation in *S. iniae* core genome genes, despite the remarkable variability in pangenome including virulence and antigenicity regions such as capsule biosynthesis genes (Lowe et al., 2007; Heath et al., 2016; Zeng et al., 2016). As a result of this, one major advantage of the use of MLST over say Multiple-Locus Variable number tandem repeat Analysis (MLVA) for example, is the use of these slowly evolving housekeeping genes (Jenke et al., 2011). This allows researchers to more reliably deduce long-term evolutionary events which may occur. However, that said, MLVA might still have higher discriminatory power and be useful to distinguish closely related serotypes or to monitor vaccine escape strains as and when needed.

### Multiple Emergence of This Pathogen on Reunion Island

Although we are unable to ascertain the exact origin of the *S. iniae* strains responsible for the epizootics on Reunion Island we are able to generate a number of hypotheses from our data. Indeed, the strains isolated during the different epizootics on Reunion Island belonged to different STs. This pattern does not support the emergence of a specific clone adapted to the local environment setup. Early strains isolated in 1996 (from a freshwater aquaculture facility) and 2002 (one of the reef fish epizootics) both belong to ST11, which also comprises three Israeli *S. iniae* strains isolated in 1989, 1998 and 2000 from Tilapia and rainbow trout aquaculture facilities (Table 1 and Figure 2). The strain isolated in 2009 on Reunion Island was obtained from diseased red drums in an offshore farm and belonged to ST12, itself a direct descendant of ST11. This suggests a common origin of these early Reunionese strains with Israeli aquaculture strains, followed by a local diversification. Even though these two countries are geographically distant, the pathogen could have been introduced to the Island *via* carrier fish import for aquaculture development on Reunion Island and/or aquarists. Israel was indeed, in the early 1990s, a major exporter of tilapia breeding lines for the growing tilapia industry worldwide (Popma and Lovshin, 1996). Unfortunately, no information regarding the origin of the diseased rainbow trout from the 1996 epizootic in Reunion, or of other fishes coexisting with them at that time in the farm, such as Tilapia, was available to confirm this hypothesis. The likely freshwater origin of the strain isolated during the 2002 epizootic is in keeping with the ecological plasticity of *S. iniae*, able to survive and adapt from a fresh water aquaculture facility around 900 meters above sea level to coastal environments, and across several host species. The example of ST8 and the probable spread of *S. iniae* in Australian barramundi, from freshwater aquaculture to sea cages tends to confirm that this hypothesis is plausible. Transmission of *S. iniae* from caged to wild fish has been suggested in the past (Colorni et al., 2002), nevertheless, to our knowledge, it is the first report of the possible spread of a pathogenic strain from freshwater aquaculture to reef fish. It is important to note that trout from the Reunionese facility affected by *S. iniae* in 1996 were later released into the rivers surrounding the farm in order to develop recreational fishing (Fédération de la pêche de La Réunion). As the first reef fish epizootic in 2002 originated from the same ST (ST11) as the one affecting trout from the freshwater aquaculture facility in 1996, this underlines the importance of developing robust and sensitive molecular tools to screen animals for pathogens before any release into the wild.

In contrast, the *S. iniae* strain involved during the last 2014 epizootic was identified as different from those isolated in 1996, 2002 and 2009 and matched the more ancestral and cosmopolitan strain, ST4. A double reversion of *Mut*L and *Mut*M would be needed to obtain the haplotype found in 2014 from the one found in 2009. This seems highly unlikely considering the very low mutation rates in DNA repair genes *Mut*L and *Mut*M (Denamur and Matic, 2006). As a consequence, we propose that this last epidemic results from the local emergence of a *S. iniae* lineage of cosmopolitan distribution.

### Tentative Commentary on Genetic Diversity of *S. iniae* Worldwide

Only 13 different STs belonging to two CCs were found within our collection of 89 strains. Moreover, a unique ST (ST4) representing 46.07% of our sample was found on all continents (North America, Australia, Asia). Clearly, the choice of critical functional genes as MLST loci will constrain diversification, as they are likely under strong purification selection. Consistently, the genetic diversity of housekeeping loci included into the scheme was low. However, this lack of variability might result from a sampling bias towards pathogenic strains from aquaculture. Indeed, it should be noted that further work on this topic including a greater range of samples may show a greater diversity in housekeeping genes. Of the 89 isolates examined, 62 are from only two species (tilapia and barramundi) and four locations. 48 originate from Australia and found in Barramundi, and 14 are from Tilapia (from Israel, United States and Hondura). That leaves only 13 strains originating from different fish species. We were also only able to map these to seven from human origin and two from dolphins. Therefore, the samples may be biased in both the host species and geographical distribution. We also did not include non-virulent strains or those solely associated with the environment as these were not available. It would be interesting to include more non-pathogenic strains in order to determine whether this lack of diversity is specific to pathogenic strains or common to environmental and/or avirulent strains, less subject to host immune selection. However, at the moment, it is still hard to isolate environmental strains. Indeed, confirmation of isolate identity as *S. iniae* by commercial bacterial identification kits is still problematic due to the biochemical profile being absent from databases supplied with the kits or variability of some atypical *S. iniae* strains (Roach et al., 2006). Moreover, most of the epizootics caused by this species occur in developing countries and remain underreported. Nevertheless, atypical strains isolated from mammals were almost always found in an ST also including strains isolated from aquaculture or ornamental fish. As such, increasing the number of host species may not necessarily increase *S. iniae’*s diversity and the results obtained in this study may constitute an informative baseline to be completed in the future to reassess the diversity of this pathogen on a broader host and geographical range.

Alternatively, we suggest that the low diversity of housekeeping genes may be due to a recent speciation of *S. iniae*, as hypothesized for other streptococci such as *S. thermophiles* (Delorme et al., 2010) or *S. agalactiae* (Brochet et al., 2006), both also known fish pathogens in their own right (Pereira et al., 2010; Bowater et al., 2012). As far as *S. agalactiae* is concerned, the commonly used MLST of Jones et al. (2003) was not sufficiently resolutive to define the population structure of the pathogen in aquatic hosts, and new typing systems were developed, including combination of MLST (sequencing of up to 15 housekeeping genes), serotyping and presence/absence of virulence associated genes (Sørensen et al., 2010; Delannoy et al., 2013; Godoy et al., 2013). These combined approaches might allow resolving the main phylogenetic events, but are time consuming and costly. The low diversity observed herein may also result from a recent bottleneck history, reducing the size and diversity of *S. iniae* populations. In many instances, genetically monomorphic pathogens have undergone such bottlenecks, for example following a crucial genetic event, causing a change in ecological niche (Achtman, 2008).

### Ecological Flexibility

The low diversity observed in the MLST seems associated to a great ecological flexibility of this pathogen. Our data confirms that *S. iniae* does not appear to have any major host specificity (Agnew and Barnes, 2007). Although most of our sampling originates from farmed fish, where it causes the highest mortalities, the few available STs obtained from infections in mammals (including humans) did not cluster together but rather with STs obtained from other fish isolates (Table 1; ST2, ST3), suggesting transmission from farmed fish to humans (Weinstein et al., 1997; Facklam et al., 2005). Genomic data indicate that a host jump might have been facilitated by rapid mutations (Silayeva et al., 2020). This ecological flexibility, underlined by the apparent lack of host specificity of *S. iniae*, is further emphasized by the ability of a particular clone to survive both in fresh and seawater. As already discussed, ST11 groups with early strains isolated from freshwater aquaculture in Israel and Reunion Island as well as the first strain responsible for a reef fish epizootic on Reunion Island. ST8 is another example of ST genotyped from barramundi freshwater farms in Western Australia (2004) and from sea cages in the Northern Territory (2005–2006; Table 1). This ST has been associated with important fish loss since 2004 in these two regions where *S. iniae* was not described before (Creeper and Buller, 2006). Although both Western Australia and Northern Territory strains were genotyped as ST8, Nawawi et al. (2009) showed that Northern Territory isolates were further characterized by a mutation on the lactate oxidase gene (*lctO*) and able to process lactate at a faster rate than the other strains (Nawawi et al., 2009). This change has been attributed to environmental influences of large tidal flows linked with increased swimming activity of the barramundi hosts in seawater, that have led to the evolution of the *lctO* gene variant, encoding a more efficient enzyme in these specific isolates (Nawawi et al., 2009). Although our MLST does not highlight this kind of rapid discrete mutation, it does enable retracing the probable common origin of isolates from different locations. Further, MLST enables the exploration of ecological flexibility of *S. iniae*, indicating the ability to switch to various hosts and habitats (freshwater vs. seawater).

## Conclusion

Although MLST based on the sequencing of a few housekeeping genes was developed almost 20years ago, it is still a rapid, convenient and relevant method to shed light on the origin and long term evolution of bacteria (Pérez-Losada et al., 2013; Jolley and Maiden, 2014). We selected eight housekeeping genes that were able to distinguish the main *S. iniae* phylogenetic clades and this enabled us to explore both origin and evolution of *S. iniae* causing mass fish die offs on the remote Reunion Island. The MLST scheme suggests at least two different origins of the pathogen causing epizootics on the island. The strains involved in 1996, 2002 and 2009 epizootics are genetically closely related and probably share a common ancestor with aquaculture strains from Israel, whereas the strain isolated in 2014 belongs to a more cosmopolitan ST. In addition, results of our MLST seemed to indicate an ecological flexibility of this pathogen, with some strains able to infect mammals as well as fish hosts or colonize both fresh and seawater environments. Despite or maybe because of this ecological flexibility, we observed a low global genetic diversity of this pathogen. That said, we cannot ignore this result may reflect a sampling bias towards aquaculture fish species most affected by this pathogen. However, we still encourage the use of this quick and convenient tool to document the diversity of this pathogen on new host species, regions, and environmental sources when available. Finally, considering the low genetic diversity of *S. iniae*, the results obtained to investigate the recurrent epizootics on Reunion Island underline the usefulness of this MLST scheme to monitor disease emergence, retrace possible transmission routes and investigate the evolution of this pathogen over recurring epizootics.

## Data Availability Statement

The datasets presented in this study can be found in online repositories. The names of the repository/repositories and accession number(s) can be found in the article/supplementary material.

## Ethics Statement

The animal study was reviewed and approved by Marine Reserve of Reunion Island.

## Author Contributions

SI and MS conceived and designed the experiments, performed the experiments, analyzed the data, contributed to reagents, materials, and analysis tools, wrote the paper, prepared figures and/or tables, and reviewed drafts of the paper. MS wrote the paper, prepared figures and/or tables, and reviewed drafts of the paper. OS, PC, and AB conceived and designed the experiments, wrote the paper, prepared figures and/or tables, and reviewed drafts of the paper. PT conceived and designed the experiments, performed the experiments, contributed to reagents, materials, and analysis tools, wrote the paper, prepared figures and/or tables, and reviewed drafts of the paper. All authors contributed to the article and approved the submitted version.

## Funding

This work was cofunded in the frame of the REMPOR project by the European Union (EU, FEDER), the Regional Council of Reunion, and the French Department of Ecology, Sustainable Development, Transportation and Housing (DEAL).

## Conflict of Interest

The authors declare that the research was conducted in the absence of any commercial or financial relationships that could be construed as a potential conflict of interest.

## Publisher’s Note

All claims expressed in this article are solely those of the authors and do not necessarily represent those of their affiliated organizations, or those of the publisher, the editors and the reviewers. Any product that may be evaluated in this article, or claim that may be made by its manufacturer, is not guaranteed or endorsed by the publisher.

## Footnotes

^1^ http://www.comparingpartitions.info

^2^ http://guanine.evolbio.mpg.de/cgi-bin/lian/lian.cgi.pl/query
